# Varieties of Economic Elites? Preliminary Results From the World Elite Database (WED)

**DOI:** 10.1111/1468-4446.13203

**Published:** 2025-03-27

**Authors:** Felix Bühlmann, Caroline Ahler Christesen, Bruno Cousin, François Denord, Christoph Houman Ellersgaard, Paul Lagneau‐Ymonet, Anton Grau Larsen, Mike Savage, Sylvain Thine, Kevin Young, Pedro Araujo, Paola Arrigoni, Jorge Atria, Pierre Benz, Johanna Behr, Maria do Carmo Botelho, Asif Butt, Pedro Casanova, Luís Clemente‐Casinhas, Ana Castellani, Fabio Cescon, Joselle Dagnes, Hanna Debska, Anne‐Sophie Delval, Vitalina Dragun, Andreia Egas, Kajsa Emilsson, Xiaoguang Fan, Fan Fu, Julia Gentile, Orlando Gomes, Victoria Gronwald, Mariana Heredia, Johannes Hjellbrekke, Jorge Honório, Jie Huang, Johnathan Inkley, Håkan Johansson, Ilkka Koiranen, Aki Koivula, Hanna Kuusela, Gabriel Levita, Chao Ling, Peng Lu, Michael Lukas, Jacob Lunding, Mina Mahmoudzadeh, Sean McQuade, María Luisa Méndez, Nuno Nunes, Shay O'Brien, Gabriel Otero, Marta Pagnini, Alejandro Pelfini, Jéssica Pereira, Catalina Roa, Thierry Rossier, Marte Lund Saga, Dulce Santana, Christian Schneickert, Elisabeth Schimpfössl, François Schoenberger, Izaura Solipa, Łukasz Trembaczowski, Maren Toft, Sofia Vale, Pedro Vasconcelos, Jorge Quesada Velazco, Tomasz Warczok, Xinguo Yu

**Affiliations:** ^1^ LIVES University of Lausanne Lausanne Switzerland; ^2^ Department of Social Sciences and Business Roskilde University Roskilde Denmark; ^3^ CEE Sciences Po Paris France; ^4^ CESSP CNRS Paris France; ^5^ Department of Organization Copenhagen Business School Copenhagen Denmark; ^6^ IRISSO Paris Dauphine University – PSL Paris France; ^7^ Department of Sociology London School of Economics and Political Science London UK; ^8^ International Inequalities Institute London School of Economics and Political Science London UK; ^9^ CURAPP Université de Picardie Jules Verne Amiens France; ^10^ CESSP CNRS EHESS Université Paris 1 Panthéon‐Sorbonne Paris France; ^11^ Department of Economics University of Massachusetts‐Amherst Amherst Massachusetts USA; ^12^ Swiss Centre of Expertise in the Social Sciences Lausanne Switzerland; ^13^ University of Lausanne Lausanne Switzerland; ^14^ Department of Political and Social Sciences University of Bologna Bologna Italy; ^15^ Institute of Sociology Pontificia Universidad Católica de Chile Santiago Chile; ^16^ Centre for Social Conflict and Cohesion Studies (COES) Santiago Chile; ^17^ School of Library and Information Sciences University of Montreal Montreal Canada; ^18^ Institute of Political Studies University of Lausanne Lausanne Switzerland; ^19^ Department of Social Research Methods Iscte‐University Institute of Lisbon Lisbon Portugal; ^20^ CIES‐Iscte Iscte‐University Institute of Lisbon Lisbon Portugal; ^21^ Department of Economics Iscte‐University Institute of Lisbon Lisbon Portugal; ^22^ Business Research Unit (BRU‐IUL) Lisbon Portugal; ^23^ Centro de Innovación de los Trabajadores (CITRA) CONICET Buenos Aires Argentina; ^24^ University of Buenos Aires Buenos Aires Argentina; ^25^ University of Amsterdam Amsterdam the Netherlands; ^26^ Department of Culture, Politics and Society University of Turin Turin Italy; ^27^ University of the National Education Commission Krakow Poland; ^28^ CREST Institut Polytechnique de Paris Palaiseau France; ^29^ Iscte‐University Institute of Lisbon Lisbon Portugal; ^30^ School of Social Work Lund University Lund Sweden; ^31^ School of Public Affairs Zhejiang University Zhejiang China; ^32^ Department of Sociology University of California San Diego La Jolla California USA; ^33^ Escuela Interdisciplinaria de Altos Estudios Sociales (EIDAES) National University of San Martin San Martin Argentina; ^34^ Department of Sociology University of Bergen Bergen Norway; ^35^ School of Government Nanjing University Nanjing China; ^36^ Department of Social Research University of Turku Turku Finland; ^37^ INVEST Research Flagship Centre University of Turku Turku Finland; ^38^ Department of Social Sciences and Philosophy University of Jyväskylä Jyväskylä Finland; ^39^ Instituto de Problemas Nacionales CONICET Lanus Argentina; ^40^ Universidad del Salvador Buenos Aires Argentina; ^41^ School of Sociology and Psychology Central University of Finance and Economics Beijing China; ^42^ Institute of Sociology Chinese Academy of Social Sciences Beijing China; ^43^ University of Chile Santiago Chile; ^44^ DACSS University of Massachusetts Amherst Amherst Massachusetts USA; ^45^ IEUT Pontifical Catholic University of Chile Santiago Chile; ^46^ Department of Social and Enterprise Sciences Iscte‐University Institute of Lisbon Lisbon Portugal; ^47^ Stone Program in Wealth Distribution Inequality and Social Policy Harvard University Cambridge Massachusetts USA; ^48^ School of Sociology Universidad Diego Portales Santiago Chile; ^49^ Facultad de Ciencias Sociales Universidad del Salvador Buenos Aires Argentina; ^50^ FLACSO Buenos Aires Argentina; ^51^ University Diego Portales Santiago Chile; ^52^ Department of Sociology and Human Geography University of Oslo Oslo Norway; ^53^ Department of Sociology University of Magdeburg Magdeburg Germany; ^54^ School of Social Sciences and Humanities Aston University Birmingham UK; ^55^ University of Silesia in Katowice Katowice Poland; ^56^ Department of Sociology Iscte‐University Institute of Lisbon Lisbon Portugal; ^57^ Centre for Research and Studies in Sociology (CIES‐Iscte) Iscte‐University Institute of Lisbon Lisbon Portugal; ^58^ Institute of Political Science University of St. Gallen St. Gallen Switzerland; ^59^ University of Warsaw Warsaw Poland; ^60^ Research Center for Private Entrepreneurs Chinese Academy of Social Sciences Beijing China

## Abstract

The strategies, decisions and beliefs of those who occupy prominent positions of economic power have influence on very large corporations and the markets they dominate, on vast amounts of economic resources, and on the rules of the game. However, the sociology of elites faces a dual challenge: divergent conceptualisations of what can be considered as a position of economic power and internationally incompatible sources of information hinder comparative analysis. The World Elite Database (WED) addresses this dual challenge, by generating, based on a consistent definition, standardised data for 16 countries. This research note introduces WED, its construction principles, and presents preliminary findings on how economic elites differ across countries.

## 1 The Challenge of Comparing Elites

Elites matter. The prominent positions they occupy in organisations and the vast resources at their disposal mean that their strategies, decisions, practices and beliefs have collective implications. Social science needs to focus on the individuals who occupy positions of power, and more specifically positions of *economic* power: defining the work conditions of thousands of employees and shaping market chances of competing firms, mobilising fortunes or elaborating the rules that organise economic activities is a crucial source of discretion within capitalist societies. The sociology of elites has become a vibrant domain within the discipline (Cousin et al. 2018), but it still faces a dual challenge. On the one hand, scholarship is most frequently about national cases studied with different theories and definitions of elites. On the other hand, the populations used to study economic elites and the type of data we have on them vary widely both between and within countries. This is why, until now, sociologists have struggled to develop a comprehensive comparative research framework on economic elites.

The World Elite Database (WED) is an international consortium of 70 social scientists founded in 2022. Coming from different countries, disciplines and schools of thought, we aim at addressing this dual challenge by generating a range of concepts, samples and variables to systematically describe, compare and explain national economic power structures (Savage 2024). Generally, we find that those who occupy positions of economic power combine the most traditional characteristics of domination—seniority, masculinity, native majority—with advanced educational credentials. However, variations across countries are not reducible to existing typologies, such as varieties of wealth, capitalism, or welfare (Beckert 2023; Hall and Soskice 2001; Esping‐Andersen 1990).

## 2 Justifying the Study Populations

Thus far WED has 16 participating countries, representing a solid starting point. First, existing comparative studies on economic elites generally analyse only a handful of countries, and often within a single region of the world.^1^ What is common to most of these studies is that they are either analyses of corporate board members, separate studies of rich lists or sectoral case studies. In so doing, existing scholarship tends to focus on one form of economic power at a time. Second, our current selection of countries contains significant institutional variation. Forms of government, levels of democratisation, gender norms, character of civil society, population size, country wealth, levels of integration in the global economy, and many other indicators vary across it.

Third, while our list is not representative, it does represent some of the largest economies and most populated countries in the world. The 16 countries currently included count for 54% of the global GDP and 33% of the global population. Among the world's billionaires, 74% live in a country part of our sample. A full table of each of these figures per country is available in Table A1. Fourth, we intend to grow the number of countries in WED over time. Part of its aim is to expand the list of country teams in the forthcoming years, bringing greater variation, coverage, and national expertise together. Indeed, this *BJS* research note is also meant to be a communication device to our peers with in‐depth country expertise who might want to join us.^2^

## 3 Identifying Positions of Economic Power

We conceive of economic power as composed of organisational power, market power and regulatory power. Organisational power refers to the power over the resources of the organisation by its operational direction or its owners (Schmalz 2018). Market power refers to companies using their size (or individuals using their wealth) to manipulate prices, discourage competition and curb labour demands (Lamoreaux 2019). Regulatory power is about holding sway over the rules of the economic game (Pistor 2019), through lobbying, political donations, lawfare or corruption, even though the latter cannot systematically be documented. Furthermore, WED's final aim is not to consider variables one by one (as we do in this research note). Rather, our goal is to enable relational analyses and cross‐country comparisons based on datasets of individual characteristics and organisational attributes that are frequently associated with positions of economic power.

WED thus adopts a positional approach (Hoffmann‐Lange 2018) based on three main distinct ways of holding economic power: (1) control over the largest corporations, (2) ownership of vast amounts of assets, and (3) ascendancy over the organisations involved in regulating the economy. What is unique about the WED country populations is that they include all three types of positions in a systematic way, but also allow for sensitivity towards their relative importance and institutional variation across countries. To grasp the three forms of economic power in a comparable way, economic elites are selected according to three identical criteria in all WED countries (for details on the construction of the sample in each country, go to https://worldelitedatabase.org/methodology).

Criteria 1 identifies those who control large corporations. Criteria 1a includes the chairpersons and chief executive officers (CEOs) of the *publicly‐listed* companies that compose the main stock index in a given country. It not only represents a top sample of large and powerful organisations, but also symbolises, rightly or wrongly, the national economy as a whole to many participants and observers. Criteria 1b identifies the chairpersons and CEOs of other very large companies, especially *privately held* or *state‐controlled* firms. Criteria 2 identifies the individuals who are listed on national rich lists. To select positions of economic power that are of similar magnitude, these criteria are related through common metrics. Criteria 1b is related to 1a by a size threshold: only firms ranked above the bottom quartiles of the first‐criterion blue chips' annual earnings in USD and of their numbers of employees at the reference date are selected. Criteria 2 is also related to 1a: only those individual and family fortunes exceeding half the average capitalisation of the three smallest companies in the main stock index are included.

Criteria 3 covers the heads of organisations who exert regulatory power. For this criterion, WED members agreed on a list of positions from which one can shape the rules of the economic game: elected politicians and top bureaucrats in charge of economic affairs (i.e., chairs of standing committees, ministers, chairs of regulatory authorities and of the central bank), interest groups (employers' representatives, lobbyists, trade unionists, some think tankers) and key intermediaries (corporate lawyers, investment bankers, audit firm partners, strategy consultants and asset managers). Through their expertise and based on contemporary literature,^3^ each national team made decisions on the relevance of the above‐mentioned categories in their national institutional arrangements. These decisions, based on whether these positions were comparable in power and status to the positions of the individuals included under criteria 1 and 2, are also documented and justified for each country in our online repository (see https://worldelitedatabase.org/methodology).

## 4 Digital Prosopography

We resort to prosopography (Bukodi and Goldthorpe 2021), collecting individual characteristics and organisational attributes that are usually associated with positions of power. This inevitably involves trade‐offs. On the one hand, we want to limit the number of individuals, as searching for individual information in different sources is time‐consuming and requires extensive case‐by‐case checking. On the other hand, we do not want too few individuals, as volume has statistical benefits. In this perspective, our criteria ensure the selection of relevant actors: all key players in the national economy are included and all included individuals hold a similar minimal amount of economic power.

The collection of information implies identifying sources that are accessible and trustworthy. WED mainly relies on public information, that is, information made publicly available by the press and companies' PR (published bios and portraits, appointment or resignation press releases), through regulatory compliance (annual reports, reference documents, company registers, statutes of incorporation), by the individuals themselves (CVs, LinkedIn profiles) or with their consent (in the case of biographical dictionaries). All this information is collected in local idioms, but coded in English and linked, when possible, to other publicly available (e.g., Wikidata) or proprietary (e.g., BoardEx and Orbis) sources. Such linkages provide great potential, but, as both E. Heemskerk et al. (2018) and Schoeneman (2022) have pointed out, many off‐the‐shelf ‘big data’ sources of elite populations can be especially problematic in light of issues such as entity resolution, data fidelity, and representativeness, and must be curated, cleaned and pre‐processed in intensive ways for the data to be meaningfully useful at large scales.

Embedding data collection in national team structures ensures that we have can make use of the best sources appropriate to a given country context, utilise a wide variety of languages, and inform our search for information in light of institutional and historical context of each country. When sources provide different information, WED members choose the most official source. A database management system facilitates the storage of information and data cleaning, as well as quality checks across countries. WED currently covers 3850 positions held by 3543 unique individuals in 16 countries. Each team undertakes to update the data for which it is responsible every 2 years, beginning in 2025—a process that we hope also encourages the addition of new national country teams over time.

## 5 Overlaps

Table 1 below breaks down the study populations by the three criteria described above in absolute numbers and percentage terms. In general, ‘overlaps’—individuals who appear through several criteria, as documented in the rightmost column below—are rare. However, forms of economic power seem less differentiated in France, Russia and China than in countries like Sweden, Finland, Poland, Germany, Switzerland, the US and the UK, while there are no overlaps in positions in Argentina. Overlaps across countries are even more exceptional: less than 1% (32 out of 3543) of the total number of individuals appear in more than one study population.

**TABLE 1:** Included positions across 16 countries and selection criteria, sorted by proportion of criterion overlap.

	Criteria 1											
Country	Criteria 1a—Main index		Criteria 1b—Other large firms		Criteria 2—Wealth		Criteria 3—Economic policy		Total positions	Individuals	Individuals in more than one criteria	
France	98	38%	26	10%	65	25%	71	27%	260	229	31	14%
Russia	128	23%	68	12%	200	36%	155	28%	551	470	66	14%
China	206	60%	49	14%	36	11%	50	15%	341	304	35	12%
Italy	78	22%	161	44%	33	9%	89	25%	361	336	26	8%
Norway	37	30%	19	15%	34	27%	35	28%	125	116	9	8%
Denmark	47	29%	52	32%	20	12%	41	26%	160	150	10	7%
Portugal	27	34%	25	32%	6	8%	21	27%	79	74	5	7%
USA	131	50%	14	5%	46	17%	73	28%	264	250	14	6%
UK	194	34%	168	30%	71	12%	136	24%	569	540	29	5%
Finland	49	37%	19	14%	7	5%	57	43%	132	127	5	4%
Germany	58	34%	39	23%	30	18%	44	26%	171	165	6	4%
Poland	37	29%	32	25%	25	20%	33	26%	127	122	5	4%
Switzerland	88	41%	48	22%	35	16%	45	21%	216	208	8	4%
Chile	58	26%	97	43%	12	5%	60	26%	227	221	6	3%
Sweden	56	39%	19	13%	28	20%	40	28%	143	139	4	3%
Argentina	29	23%	64	52%	10	8%	21	17%	124	124	0	0%
Total	1321	34%	900	23%	658	17%	971	25%	3850	3575^a^	259	7%

^a^ The total number of individuals is in fact 3543 as 32 individuals are part of at least two WED populations.

The differences in national population numbers are driven by the fact that, across the three criteria, there are a different number of firms in the main stock index, a different number of large‐enough non‐listed firms, a different array of super‐rich individuals, and different institutional arrangements in terms of regulating the game of the field of economic power. Additionally, some organisations have a dual leadership structure, for example, with a chair and a CEO, rather than a singular leader. These international variations within criteria signal varieties of national political economies (Amable 2003).

## 6 Age

**FIGURE 1:**
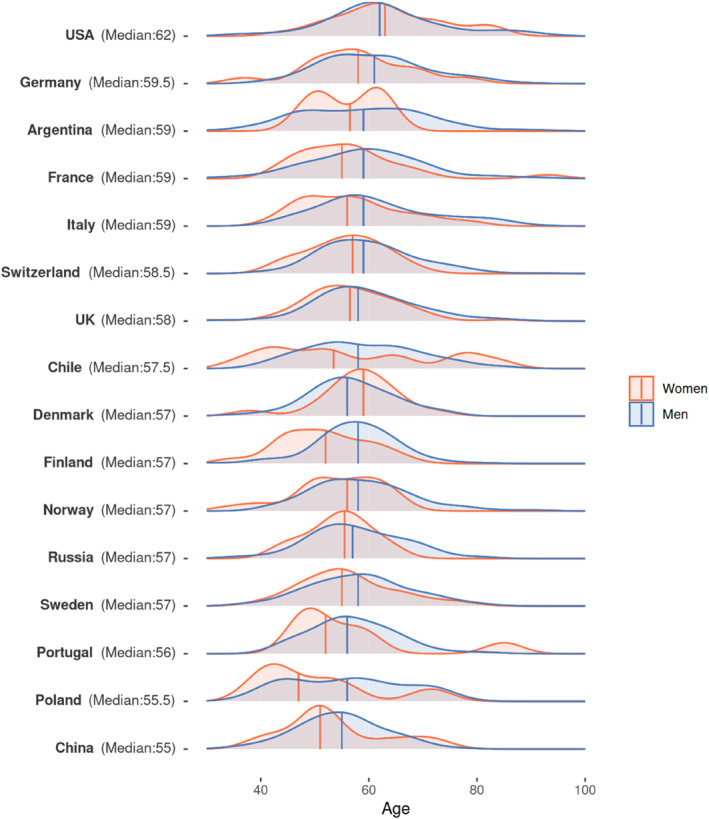
Age distribution by country and gender, sorted by median age.

Findings regarding age suggest that the gerontocratic norm prevails. Nonetheless, there are significant variations between countries, as Figure 1 below makes clear. The US economic elite stands out as the ‘oldest’, with a median age of 62 years. Most European countries are quite similar to each other in terms of median age of their WED population, ranging from 59.5 (Germany) to 57 (Finland). The youngest economic elites are found, notably, in China and Poland, where a significant share (7%) of the national WED population is under 40. These are also the two only countries where individuals included under criterion 3—the regulators of the game—are the oldest subgroup. It is plausible that the age of economic elites might be related to the timing of integration of their country in the (post‐Cold War) world economy or to its position at either the core or the periphery of the global network of corporate interlocks and career hubs (E. M. Heemskerk and Takes 2016; Bühlmann et al. 2024).

One could assume that women in the WED populations needed to be older than their male counterparts, as they might have to take longer to accumulate a larger amount of several forms of capital to ‘compensate’ the gender bias. However, only in Denmark and the US are women economic elites older than men. Elsewhere, men are more senior than women, ranging from a couple of years—in Russia, Norway, Switzerland and the UK—to 9 years in Poland. This suggests that gendered selection patterns into elite populations differ in systematic ways in nearly all countries.

## 7 Gender

**FIGURE 2:**
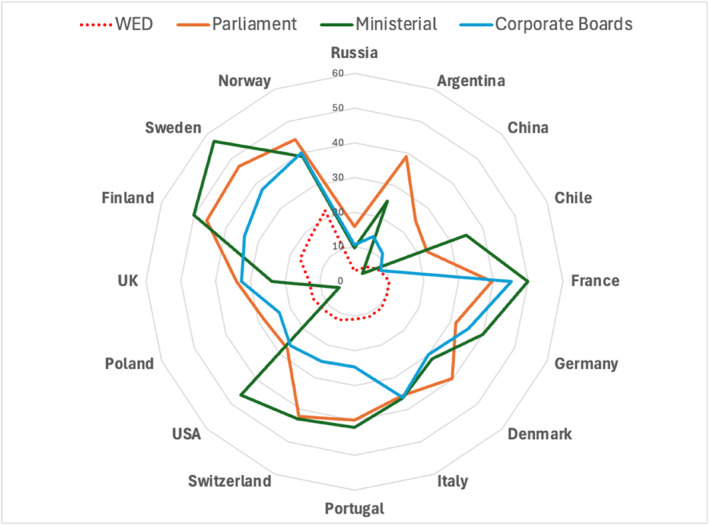
Spider plot of gender ratios.

It is not surprising that the economic elites are predominantly male. Nevertheless, compared to measures of women's representation in other areas of the field of power and to a larger population of executives, top economic elites are significantly far behind. Figure 2 below offers a simple comparative picture of just how male the WED population is. It visualises the simple gender ratios within national parliaments (in blue) and within the boards of publicly listed corporations (in orange), using data from international surveys conducted by the World Economic Forum (World Economic Forum (WEF) 2021), against the WED gender ratios (in green) for each country.

**FIGURE 3:**
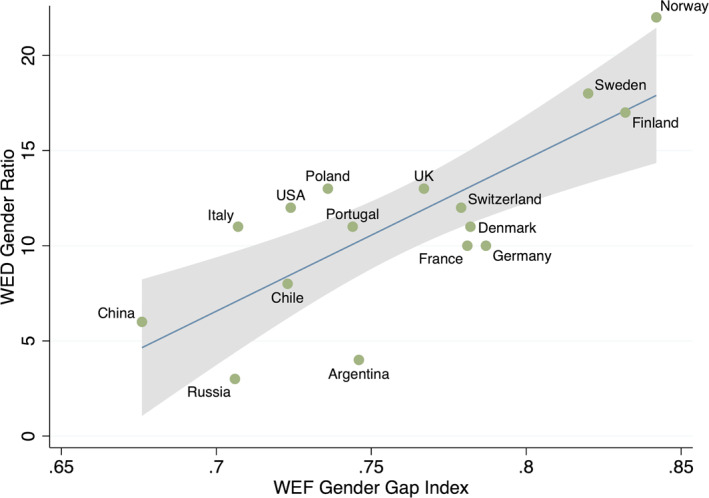
WED gender ratios and the WEF Gender Gap Index.

Interestingly, the variations in the WED gender ratios track other measures of gender equality. For instance, there is a strong, positive relationship between the number of women in the WED economic elite populations and the highly publicised World Economic Forum's Gender Gap Index (see Figure 3 below).^4^

## 8 Place of Birth

**FIGURE 4:**
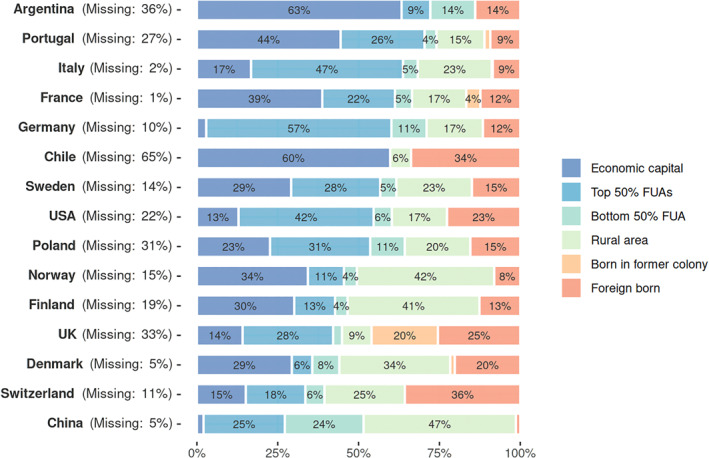
Place of birth by functional urban areas (FUAs), sorted by proportion from economic capital or top 50% FU. Russia is excluded from this analysis because of lack of data availability.

Most economic elites are born in the country where the organisation they lead is based. In spite of the globalisation of capitalism, their trajectories remain very much national (see Figure 4). However, the proportion of foreign‐born varies widely across countries: from almost none in China (1%) and Russia (6%), up to around a fourth or a fifth for the US and Denmark, and even more in Chile (34%), Switzerland (36%) and the UK (45%). Interestingly, the latter four countries correspond to different types of political economies (Hall and Soskice 2001). However, in these four cases, direct State interventionism in corporate governance is generally low. In addition, Switzerland combines a business‐friendly environment with a multicultural federal constitution, while the imperial past matters for the UK. The US likely attracts foreigners due to its central position in global capitalism, and Denmark has increasingly become an export‐led economy driven by multinational corporations.

Detailing the distribution of birthplaces within a given country using the EU‐OECD definition of functional urban areas (FUAs) and singling out the metropolis with the largest stock exchange as the economic capital, Figure 4 also shows that the natives of the latter are overrepresented in most cases.

Germany and the UK are outliers to this overall pattern. Germany because of its economic multipolarity: Frankfurt, where the main national stock exchange is located, barely makes it to the top five of largest German metropolises. Compared to the UK, France and Portugal are characterised by less elite trajectories originating in their former colonial possessions, and a clearer prominence of their capital cities. Finally, it is worth noting how countries with a diversity of important metropolitan or regional economic clusters, like Germany, Italy (Becattini 2004) or the US (Storper 1997), also tend to have a substantial share of their business leaders coming from these clusters.

## 9 Education

**FIGURE 5:**
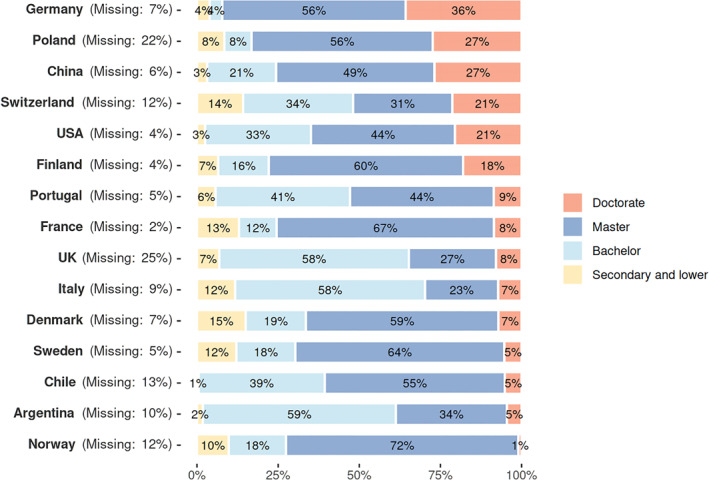
Highest attained level of education (sorted by proportion of doctorates). Russia is excluded from this analysis because of lack of data availability.

In all countries covered by WED so far, the exercise of economic power falls to individuals with higher education qualifications than the working age population. Figure 5 shows the highest degrees attained by economic elites in each country, by proportion of the economic elite population in each country.

As Figure 5 illustrates, the most common level of qualification varies from country to country: while a bachelor's degree is sufficient in Argentina, Italy and the UK, the master degree is modal in the other countries (although the difference between the two is not always easy to discern for the individuals who completed their studies before the beginning of the 21st Century and the implementation in Europe of the Bologna Process of standardisation). In France, most of the master's degrees correspond to *grandes écoles* diploma. With over a quarter of the economically powerful holding a doctorate (especially in economics or law), Germany, Poland and China stand out.

The highest graduation rates and levels are observed among individuals selected under criteria 1a, 1b and 3, which correspond to bureaucratic organisations: publicly‐listed companies, private, mutual or state‐controlled companies and official entities. Conversely, it is the second criterion (wealth, which can be mainly inherited) that displays the lowest percentages of academic credentials: on average 59% with a tertiary education, while this proportion respectively reaches 90%, 79% and 88% for criteria 1a, 1b and 3.

**FIGURE 6:**
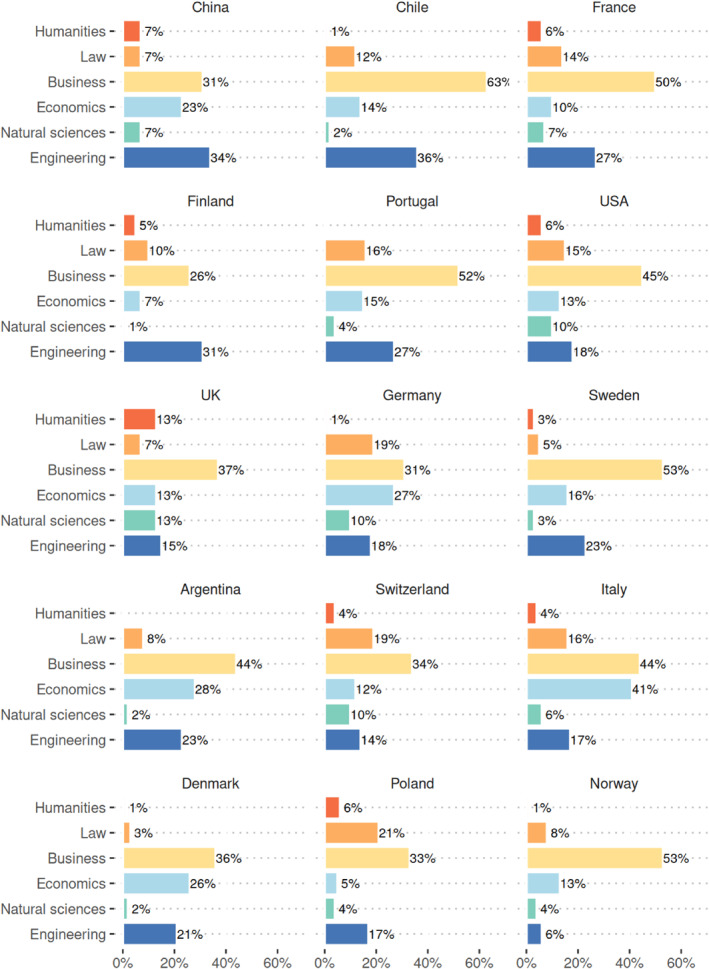
Disciplines of full‐time educational degrees (includes multiple degrees), sorted by proportion with a STEM degree (engineering or natural sciences). Russia is excluded from this analysis because of lack of data availability.

Figure 6 represents a simple breakdown of the educational degrees attained by the economic elite population across countries, based on the categories of Humanities, Law, Business, Economics, Natural Sciences and Engineering degrees, respectively. In terms of the disciplines studied, business, engineering and economics predominate. However, in the UK, Poland and Switzerland, law and the humanities account for more than 20%, while they are rare (less than 10%) in Denmark, Norway and Sweden. Only in China and Finland educational backgrounds in business are not the most common ones. Indeed, the economic elites of these countries are more oriented towards technical skills. In addition, for every national case studied here, only a small number of local or internationally recognised institutions offer these educational credentials.

## 10 Discussion

The WED is a novel attempt to define economic power—understood as organisational, market, and regulatory power—and to convert these forms of power into a series of standardised selection criteria which allow us to compare national economic elites. Since the three criteria are not mutually exclusive, WED is better equipped than ‘silo‐studies’ (‘The corporate leaders’, ‘The top bureaucrats’) to analyse the asymmetric interdependencies between business, politics and administration across countries. Currently the WED is based on 16 national cases from four continents which comprise significant institutional variation and it also includes some of the largest and most powerful economies.

Our preliminary findings show for instance how international variation in the recruitment patterns of the economic elite is not entirely straightforward. The country most open to women economic elites—Norway—is also among the most closed to foreign recruitment and doctorates. Second, while elites in countries such as Germany have very high educational credentials, the role played by the world's most prestigious universities seems marginal. A third example concerns the case of China: its economic elite is by far the youngest, but also the most male‐dominated and native‐born. Lastly, while the US elite is very old, on most other parameters it is not particularly divergent from the ones of other countries.

As with all attempts to empirically define and categorise elites (and social groups in general), our research strategy also has limits. For instance, the positional approach is ill‐suited to identify the economically powerful in countries with a large informal sector. We see that the link between criteria 1a and 1b, designed to account for the magnitude of financial markets and to recognise that not all very large companies are listed, makes it difficult to account for countries in which the private sector heavily relies on SMEs (as for instance in Germany).

Still, our first descriptive findings show that there are some intriguing differences which need further investigation. These relate especially to the geographical origins of elites, and to the way educational credentials operate. We expect to analyse educational patterns in greater detail, to link gender ratios to a variety of national institutions, or to examine the participation of our national elite populations in global policymaking forums. We also intend to use our populations to compare across countries the configurations of the national networks they constitute and of the fields of economic power. We also hope that our data can help to inform and contextualise future qualitative exploration of country‐cases or industries. In this spirit, we currently venture to expand our list of country teams and encourage colleagues from across the globe to reach out to engage with our work as it develops. A second wave of data collection, aimed at updating our material and introducing the possibility of longitudinal analysis, will start by the end of 2025 and should facilitate the inclusion of new national cases.

## Conflicts of Interest

The authors declare no conflicts of interest.

## Data Availability

The authors have nothing to report.

## Endnotes

^1^
Windolf (2002) for example, compares corporate networks across 6 countries (France, Germany, Netherlands, Switzerland, UK and USA), and Cardenas (2016) across 5 (Brazil, Chile, Colombia Mexico, and Peru), whereas Stokman et al. (1985) compare 10 and David and Westerhuis (2014), 14. Some studies have a higher number of countries considered. However, like the aforementioned, they are intra‐regional comparisons. For instance, Van Veen and Kratzer (2011) examine 15 *European* countries. Cárdenas (2012) is a notable exception in this regard, analysing 12 different countries on four continents: Australia, Canada, France, Germany, Italy, Japan, Netherlands, Spain, Sweden, Switzerland, UK and USA. Other comparative studies have focussed on elites occupying one specific role in large corporations (Maclean et al. 2006; Bühlmann et al. 2018), or compared top business leaders with billionaires (Hartmann 2018) or top political officials (Schneickert 2018).

^2^
Interested parties can contact the WED Secretariat and learn more about the initiatives under way at https://worldelitedatabase.org.

## Appendix A

**TABLE A1:** Proportion of the world's GDP, population, and billionaires across WED16 countries.

Country	GDP	Population	Number of billionaires	Billionaire wealth
Argentina	0.45	0.59	0.18	0.12
Chile	0.30	0.25	0.33	0.33
China	17.16	18.4	25.3	22.78
Denmark	0.41	0.08	0.36	0.47
Finland	0.32	0.07	0.25	0.13
France	3.09	0.85	1.52	3.91
Germany	4.54	1.08	4.94	4.78
Italy	2.22	0.78	1.85	1.56
Norway	0.43	0.07	0.44	0.29
Poland	0.70	0.49	0.29	0.16
Portugal	0.27	0.13	0.07	0.06
Russia	1.74	1.91	4.28	4.48
Sweden	0.64	0.14	1.49	1.39
Switzerland	0.87	0.11	1.45	1.11
United Kingdom	3.15	0.87	2.03	1.63
United States	24.92	4.32	26.28	33.62
Total	61.21	30.14	71.06	76.82

*Source:* GDP data is derived from World Bank based on country GDP divided by World GDP, in current US dollars, for the year 2020. Population values are taken from the percentage of world population of each country for the year 2020, from the International Monetary Fund. Billionaire population by country and billionaire wealth values are both derived from Forbes global billionaires list estimated at March 2021, using the citizenship values for each country, as opposed to residence.
